# *Salmonella* Typhimurium and *Vibrio cholerae* can be transferred from plastic mulch to basil and spinach salad leaves

**DOI:** 10.1016/j.heliyon.2024.e31343

**Published:** 2024-05-18

**Authors:** Luke Woodford, Rosie Fellows, Hannah L. White, Michael J. Ormsby, Richard S. Quilliam

**Affiliations:** Biological and Environmental Sciences, Faculty of Natural Sciences, University of Stirling, Stirling, FK9 4LA, UK

**Keywords:** Enteric pathogens, Food safety, Plastic pollution, Plastisphere, Phyllosphere, Ready-to-eat crops

## Abstract

Plastic pollution is increasingly found in agricultural environments, where it contaminates soil and crops. Microbial biofilms rapidly colonise environmental plastics, such as the plastic mulches used in agricultural systems, which provide a unique environment for microbial plastisphere communities. Human pathogens can also persist in the plastisphere, and enter agricultural environments via flooding or irrigation with contaminated water. In this study we examined whether *Salmonella* Typhimurium and *Vibrio cholerae* can be transferred from the plastisphere on plastic mulch to the surface of ready-to-eat crop plants, and subsequently persist on the leaf surface. Both *S.* Typhimurium and *V. cholerae* were able to persist for 14 days on fragments of plastic mulch adhering to the surface of leaves of both basil and spinach. Importantly, within 24 h both pathogens were capable of dissociating from the surface of the plastic and were transferred onto the surface of both basil and spinach leaves. This poses a further risk to food safety and human health, as even removal of adhering plastics and washing of these ready-to-eat crops would not completely remove these pathogens. As the need for more intensive food production increases, so too does the use of plastic mulches in agronomic systems. Therefore, there is now an urgent need to understand the unquantified co-pollutant pathogen risk of contaminating agricultural and food production systems with plastic pollution.

## Highlights

• *S.* TyphiMurium and *V. cholerae* persist on plastics adhering to leaves for 14 days.
• Transfer of pathogens from the plastisphere to the leaf occurs within 24 h.
• Fragments of plastic mulch can facilitate pathogen transfer to spinach and basil.

## 1 Introduction

Plastic pollution caused by the breakdown of mulching plastics, together with microplastic fragments in fertilisers and seed dressing, are becoming a global issue for agricultural systems [[1], [2], [3]]. Microplastics in surface waters, e.g., from wastewater discharge, can also be transferred into agricultural systems via flooding events or irrigation with contaminated water [4]. Plastics in the environment rapidly become colonised by microbial biofilm, termed the ‘plastisphere’, with such communities often containing human pathogens [5]. Additionally, livestock frequently ingest plastics in their feed [6,7], with the potential for these plastics to become colonised by enteric zoonotic pathogens during passage through the gut before subsequent delivery into agricultural systems via excretion or application of fertilisers, manure, and slurry [8,9].

Once in agricultural environments, enteric bacterial pathogens like *Salmonella enterica* can contaminate the surfaces of crops (e.g., through irrigation or rain-splash), and once on the leaf surface often express specific adhesive factors like fimbrial and nonfimbrial adhesion factors that allow them to bind to the surface of leaves or fruit [10]. *S. enterica* can multiply in the phyllosphere and continue to survive on harvested leaves of ready-to-eat crops [11], but it can also become internalised via stomata on the leaf surface [12] or through root uptake from contaminated soil [13]. Cholera infections from *Vibrio cholerae* have long been associated with contaminated foods, including fruit and vegetables washed in contaminated waters or handled by infected users with poor hygienic practices [14]; however, relatively little is known about how *V. cholerae* associates with leaf surfaces or how it interacts with the phyllosphere.

Although *S. enterica* can persist on plant leaf surfaces, this is usually at fairly low concentrations due to a limited availability of nutrients, rather than pathogen fitness [15]. Once on the surface of leaves, human pathogens face a range of environmental stressors, including UV, desiccation, and extremes of temperature. In addition, human pathogens may also be exposed to plant-specific defence responses, and antagonistic interactions with the phyllosphere community [16,17]. Subsequent internalisation of human pathogens, such as *Salmonella* spp., into the leaves of ready-to-eat crops poses a significant public health concern as washing or treating the leaf surface, e.g., with ethanol, cannot remove the pathogen before human ingestion [18].

Both *S. enterica* Serovar Typhimurium and *V. cholerae* are globally responsible for considerable rates of infection, and so their transfer to ready to eat crops has significant implications for human health. *S*. Typhimurium can cause systemic infections and together with other *Salmonella* infections, accounts for ∼594 000 cases and ∼79 000 deaths a year [19]. *Vibrio cholerae* can cause diarrheal infections, typically through the ingestion of contaminated water and is responsible for an estimated 2.9 million cases and 95 000 deaths globally annually, with the biotype El Tor responsible for the current pandemic [20]. The majority of *Salmonella* and cholera cases occur in low- and middle-income countries (LMICs), typically through poor sanitation and contaminated wastewater [21]. Importantly, plastic pollution in LMICs is increasing and becoming a major component of urban dump sites, with high potential for subsequent transfer into agricultural and food producing systems [22].

The role of microplastics in agricultural systems has thus far focused on plant health and growth, or on the disruption of microbial interactions in the rhizosphere and phyllosphere [[23], [24], [25], [26]]. However, *S.* Typhimurium can survive in the plastisphere of microplastics in agricultural soils and importantly can dissociate from the plastic surface into the surrounding soil and leachate (Woodford et al., 2024, unpublished). Although microplastics can adhere to plant surfaces and are difficult to remove by washing [27], the ability of microplastics to act as a vector for transferring human pathogens onto crop plants remains unknown [28]. Therefore, in this study, we aimed to (1) quantify the survival of *S*. Typhimurium and *V*. *cholerae* on microplastic mulch adhering to leaves of ready-to-eat crops (basil and baby spinach); and (2) determine whether these pathogens could dissociate from the plastisphere and be transferred onto the surface of leaves and subsequently persist in the phyllosphere.

## 2 - Methods

### 2.1 Bacterial strains

A pathogenic strain of *Salmonella enterica* Serovar Typhimurium (MLST ST313, D23580) encoding chloramphenicol resistance, and a toxigenic strain of *Vibrio cholerae* VO6/009 (Serotype O1; El Tor biotype) were used throughout this study. *S*. Typhimurium was grown with chloramphenicol (25 μg/ml) on Luria-Bertani (LB) agar, or in broth at 37 °C and shaken at 120 rpm, unless otherwise state, and *V. cholerae* grown on Thiosulfate–citrate–bile salts–sucrose (TCBS) agar, at 37 °C.

### 2.2 Biofilm generation and inoculation of plastic and organic substrates

Fragments of 2 × 4 mm (total surface area = 16 mm^2^) of low-density polyethylene (LDPE) microplastics were cut from black bags with a razor blade, and from the leaves of the grass *Holcus lanatus* (after being dried at 40 °C for 2 h), which is referred to hereafter as ‘hay’. Plastic and hay fragments were placed in separate 50 ml Falcon tubes containing 30 ml river water (previously screened for chloramphenicol resistant bacteria and *V. cholerae*) with additional trace metals (added as a 1 ml/L solution of CuCl2·2H_2_O, 15 mg/L; NiCl2·H_2_O, 25 145 mg/L; Na2MoO4·2H_2_O, 25 mg/L; ZnCl2, 70 mg/L; MnCl2·4H_2_O, 100 mg/L; CoCl2·6H_2_O, 120 146 mg/L; FeCl3, 4 g/L; EDTA, 2 g/L; HCl [25 %], 6.5 ml/L) [29]. Tubes were placed on a shaker at 100 rpm and left at ambient room temperature (ca. 18–21°C) for 96 h to allow a natural biofilm to develop on the surfaces of both the plastic and hay fragments. Overnight cultures of *S.* Typhimurium or *V. cholerae* were spun down at 4000 rpm for 5 min, resuspended in 20 ml phosphate buffered saline (PBS), and 300 μl of each was added into the Falcon tubes containing either plastic mulch or hay pieces (to give a final concentration of ca. 10^6^ CFU/ml). After a further 72 h, all plastic and hay fragments were removed and rinsed in Milli-Q water (Millipore Milli-Q Synthesis System, USA) to remove lightly attached microbes not part of the plastisphere biofilm.

### 2.3 Experimental excised leaf set up

Approximately similarly sized leaves from fresh basil (*Ocimum basilicum*) plants were excised at the petiole, and leaves of commercially available pre-packaged baby-leaf spinach (*Spinacia oleracea*) with no visible damage were selected. Single leaves of each plant were placed in replicate 90 mm Petri dishes directly onto cotton pads soaked in 25 ml Milli-Q water. Twenty pieces of contaminated LDPE plastic or hay were added to either the adaxial or abaxial surface of replicate leaves. To act as a ‘culture-only’ control, 20 μl of a diluted overnight culture of *S.* Typhimurium or *V. cholerae* was pipetted directly onto the surface of the leaf, and the area circled with a fine marker. All Petri dishes were left at room temperature (17–22 °C) in natural light (no direct exposure to sunlight) in a glasshouse, with the lids on. No additional water was added to the dishes after the initial set up.

**Fig. 1:**
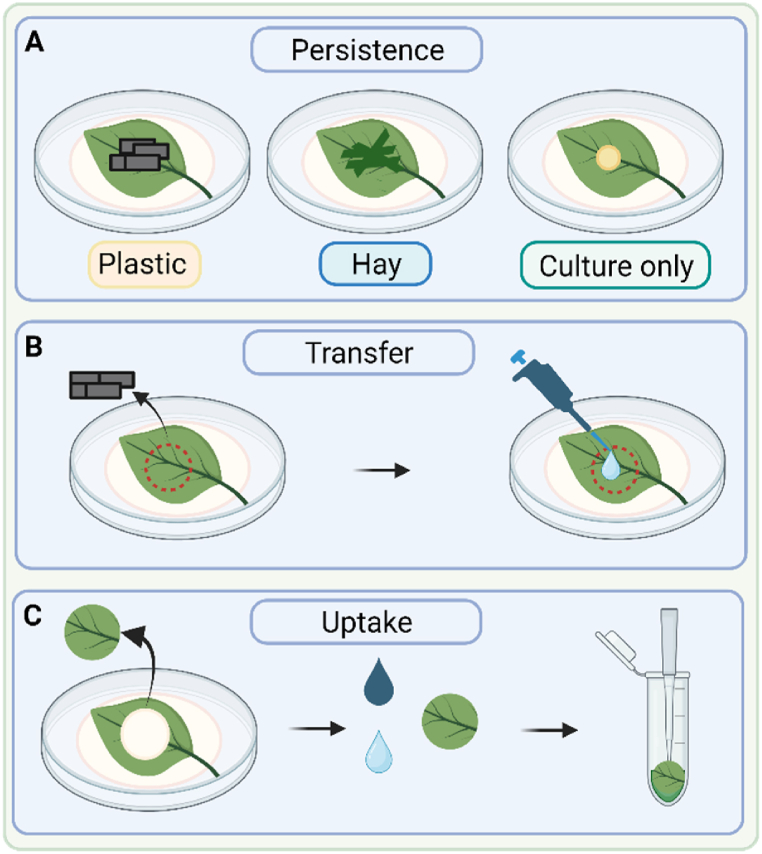
Experimental set-up to quantify, (A) persistence of *S*. Typhimurium and *V. cholerae* on the surfaces of plastics or hay adhering to the leaf surface; (B) dissociation of *S*. Typhimurium and *V. cholerae* from the plastisphere that had become loosely bound to the surface of the leaf; and (C) any *S*. Typhimurium and *V. cholerae* that had become tightly bound or internalised into the leaf.

At each sampling timepoint, the plastic and hay fragments were removed from five replicate adaxial and five replicate abaxial basil and spinach leaves using sterile forceps and placed into sterile 30 ml glass universal tubes containing 1 ml PBS and five 5 mm glass beads (Fig. 1A). At time point 0 the plastic, hay and culture was analysed prior to being placed on the surface of the leaves. The glass universal tubes were vortexed for 30 s at 1500 rpm to disrupt the biofilm. Due to low recovery rates of *Vibrio cholerae*, plastic samples were enriched in 5 ml of alkaline peptone water (APW, Sigma Aldrich, UK) in sterile 30 ml glass universals and incubated at 37 °C for 24 h.

Immediately following the removal of the plastic and hay fragments from the leaf surface, 100 μl PBS was pipetted directly on the surface of the leaf where the fragments had been. The PBS was pipetted up and down five times to remove any loosely attached epiphytic *S.* Typhimurium and *V. cholerae* which had dissociated from the plastisphere on to the leaf surface (Fig. 1B). The same technique was used to remove the ‘culture-only’ control samples from the area marked on the leaf surface. *S.* Typhimurium or *V. cholerae* cells tightly bound to the leaf surface (or endophytically internalised) were quantified by using a steel hollow leather punch (Amazon, UK) to cut a 2.8 cm^2^ disc from the leaf where the plastic or hay fragments had been. Each replicate disc was washed three times with 300 μl 70 % ethanol and then three times with 300 μl sterile PBS to remove any remaining loosely bound cells. Leaf discs were then added to individual sterile 1.5 ml Eppendorf tubes containing 300 μl PBS and homogenised using a sterile pipette tip (Fig. 1C). The concentrations of *S.* Typhimurium and *V. cholerae* on the plastic and hay fragments, and on the leaf surface and leaf tissue were quantified on plates containing either LB (with chloramphenicol) or TCBS, incubated overnight at 37 °C, and colony forming units (CFU) enumerated.

### 2.4 PCR confirmation of *S.* Typhimurium and *V. cholerae*

Throughout the experiment, PCR confirmation was conducted on a selection of presumptively identified colonies of *S.* Typhimurium and *V. cholerae* using specific primers for *S.* Typhimurium (ttr; encoding tetrathionate reductase) and *V. cholerae* (*ompW* and *ctxA;* encoding an outer membrane protein and cholera toxin, respectively) using previously described primers and methods [30,31]. Briefly, reactions consisted of a 2× master mix (Qiagen, Germany), 0.4 μM forward and reverse primer and a single colony, made to a final volume of 25 μl using nuclease-free water. Cyclic conditions of 95 °C for 1 min, 60 °C for 1 min and 72 °C for 1 min were run for 30 cycles, with an initial 5 min at 95 °C and final extension of 72 °C for 5 min. All samples were analysed by 1 % gel electrophoresis using GelRed® staining (Biotium, USA) and visualised with UV light (data not shown).

### 2.5 Statistical analyses

Comparisons between adaxial and abaxial epiphytic and endophytic *S.* Typhimurium and *V. cholerae* concentrations were assessed by two-way analysis of variance (ANOVA) and Šidák's multiple comparisons test (GraphPad Prism Software v. 9.5.1).

To calculate *S.* Typhimurium and *V. cholerae* die-off rates linear regression was calculated as previously described [30,31]. Briefly, CFU concentrations were normalised by transforming to log_10_ CFU/leaf or log_10_ CFU/ml and a log linear regression model was fitted to the log_10_ transformed data, which is described by the equation:

Log_10_(C) = Log_10_ (C_0_) – kt

where C_0_ is the cell concentration at t = 0 and k is a die-off rate constant (day minus one).

The percentage decrease in concentration per unit time was determined as constant using the log linear model. Following a log-linear die-off profile, decimal reduction times (D-values; the number of days to reduce viable bacteria by 90 %) were calculated based on the decline rates for each population of *S.* Typhimurium or *V. cholerae*. From the linear decline rate analysis, a Pearson correlation coefficient was used to investigate the linear correlation between the K-value and D-value. Based on this, 95 % confidence ellipses were calculated and generated using R [32,33].

## 3 - Results

### 3.1 Persistence of *S*. Typhimurium and *V. cholerae* on plastic mulch on the leaf surface

**Fig. 2:**
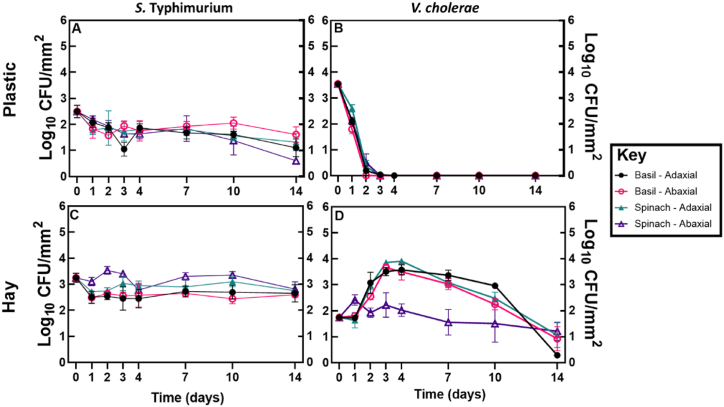
Persistence of *S*. Typhimurium and *V. cholerae* on fragments of plastic mulch (A and B) or hay (C and D) fragments adhering to the surface of basil and spinach leaves. The X-axis represents the time since the plastic or hay fragments were placed on the leaf surface. Data points represent the mean of five replicates ±SEM.

*S*. Typhimurium persisted on plastic and hay fragments on both adaxial and abaxial surfaces of basil and spinach leaves for the 14-day duration of the study. The concentration of *S.* Typhimurium on the plastic fragments at day 14 had decreased on average from 3.6 × 10^2^ CFU/mm^2^ to 2.3 × 10^1^ CFU/mm^2^, whilst the decrease on hay was from 1.8 × 10^3^ CFU/mm^2^ to 5.1 × 10^2^ CFU/mm^2^ during the same period (Fig. 2). *V. cholerae* persisted on the hay fragments for the duration of the study, decreasing on average from 5.9 × 10^1^ CFU/mm^2^ to 2.6 × 10^1^ CFU/mm^2^ by day 14; however, by day 3 the concentration of *V. cholerae* on the plastic fragments had decreased from 3.8 × 10^3^ CFU/mm^2^ to below the limit of detection. Therefore, to examine if *V. cholerae* had entered a viable but non-culturable (VNBC) state, from day 4 onwards the plastic fragments were enriched in alkaline peptone water and *V. cholerae* subsequently recovered in samples at each remaining time point (data not shown). The D-value (the days taken to reduce viable bacteria by 90 %) for *S.* Typhimurium and *V. cholerae* bound to the biofilm on the hay was higher than when the pathogens were pipetted directly onto the leaf. Linear decline analysis for *S.* Typhimurium on plastic also demonstrated considerably higher D-values compared to the culture-only control, although the D-value for *S.* Typhimurium on the surface of plastic was lower than on the surface of hay (Table 1, Figure S1). D-values for the survival of *V. cholerae* on the surface of plastic could not be calculated as the concentration had gone below the limit of detection by day 3.

**Table 1:** Linear decline rates and decimal reduction times for *S*. Typhimurium and *V*. *cholerae* recovered from plastic, hay, and culture-only controls. Linear decline rate constant = 2.303 × slope gradient (calculated from log transformed values in Fig. 2).

			*K* (day^−1^)		D-value (days)	R^2^
			Mean	SEM		
S. Typhimurium						
Basil	adaxial	Plastic	0.151	0.013	15.7	0.366
		Hay	0.018	0.018	278.4	0.068
		Culture	0.612	0.088	4.0	0.624
	abaxial	Plastic	0.061	0.019	68.3	0.145
		Hay	0.050	0.011	55.8	0.146
		Culture	0.522	0.220	8.0	0.536
Spinach	adaxial	Plastic	0.126	0.027	22.3	0.055
		Hay	0.029	0.009	121.8	0.109
		Culture	0.292	0.039	8.4	0.546
	abaxial	Plastic	0.244	0.025	9.9	0.106
		Hay	0.046	0.016	56.2	0.104
		Culture	0.525	0.028	4.4	0.664
*V. cholerae*						
Basil	adaxial	Hay	0.193	0.023	12.8	0.115
		Culture	0.840	0.105	3.0	0.310
	abaxial	Hay	0.175	0.044	18.2	0.134
		Culture	1.023	0.113	2.4	0.513
Spinach	adaxial	Hay	0.126	0.038	25.7	0.073
		Culture	1.245	0.049	1.9	0.824
	abaxial	Hay	0.225	0.116	46.7	0.279
		Culture	1.224	0.017	1.9	0.699

### 3.2 Transfer of *S*. Typhimurium and *V. cholerae* from the plastisphere to the phyllosphere

**Fig. 3:**
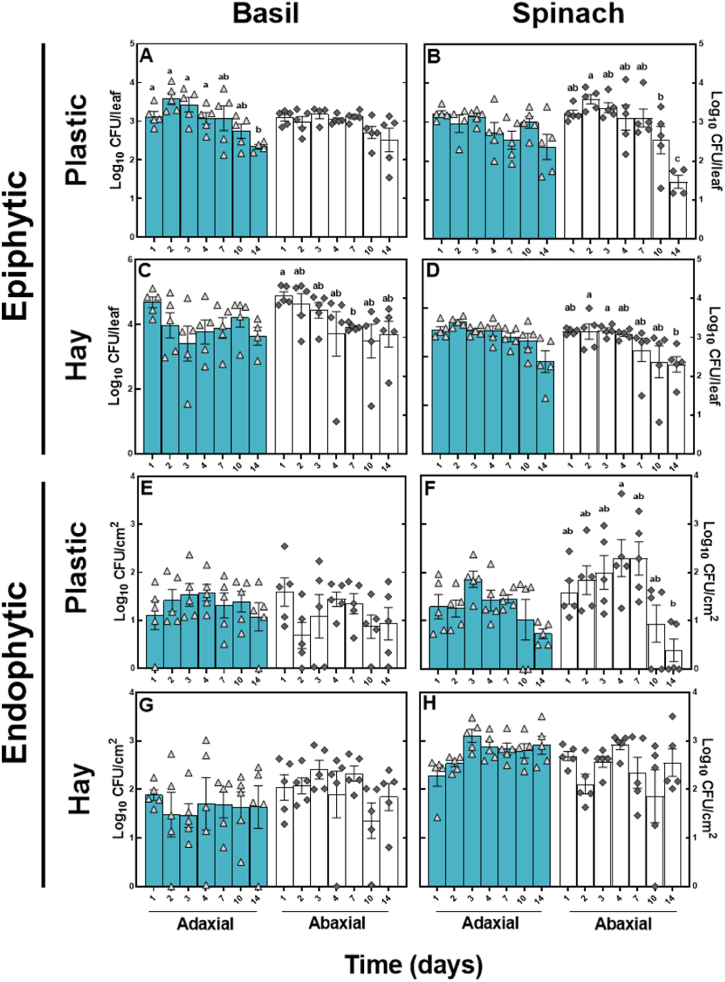
Transfer to the leaf surface and potential internalisation of *S*. Typhimurium on the adaxial and abaxial leaf of basil and spinach. *S*. Typhimurium was enumerated from a wash of the leaf surface after the removal of plastic or hay fragments (A–D) and from the same leaves homogenised after washing with ethanol (E–G). The x-axis represents the time since the plastic or hay fragments were placed on the plant leaf surface. All data is log transformed and each bar represents the mean of five replicates ±SEM. Different letters above the bars indicate significant differences (P < 0.05). All concentrations of tightly bound or endophytic cells are reported as CFU/cm^2^, calculated from a 2.8 cm^2^ standardised disk cut from each leaf.

*S.* Typhimurium colonising fragments of either plastic or hay were able to dissociate from the biofilm and become epiphytically attached to the surface of basil and spinach leaves within 24 h (Fig. 3A–D). The concentration of *S*. Typhimurium on the surface of the leaves of basil and spinach with plastic mulch on them decreased significantly on both the adaxial and abaxial surface (P < 0.05) over the 14-day period. The concentration of *S*. Typhimurium on basil and spinach leaves with hay fragments on them decreased significantly over time on the abaxial side of both the basil and the spinach (both P < 0.05), but not the adaxial side of the leaves of either. Following transfer from the plastic fragments, tightly bound, or endophytic, *S.* Typhimurium remained present throughout the 14-day period (Fig. 3E–H); however, there were no significant differences between concentrations of *S*. Typhimurium associated with the adaxial and abaxial leaf surfaces at each time point.

**Fig. 4:**
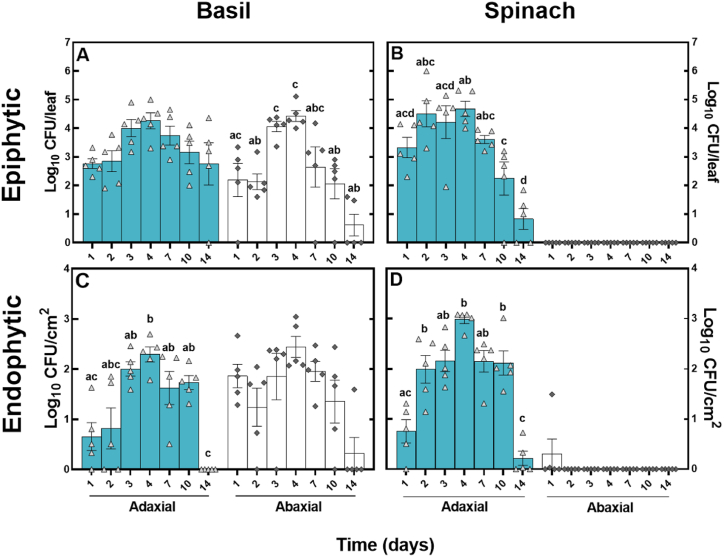
Transfer from hay to the leaf surface and potential internalisation of *V. cholerae* on the adaxial and abaxial leaf of basil and spinach. *V. cholerae* was enumerated from a wash of the leaf surface after the removal of hay fragments (A and B) and from the same leaves homogenised after washing with ethanol (C and D). The x-axis represents the time since the plastic or hay fragments were placed on the plant leaf surface. All data is log transformed and each bar represents a mean of five replicates ±SEM. Different letters above the bars indicate significant differences (P < 0.05). All concentrations of tightly bound or endophytic cells are reported as CFU/cm^2^, calculated from a 2.8 cm^2^ standardised disk cut from each leaf.

*V. cholerae* was recovered from the surface of basil leaves with adhering hay fragments for the whole 14 days (Fig. 4), whilst tightly bound and endophytic cells were recovered for the first 10 days. Concentrations of epiphytic *V. cholerae* cells on the abaxial surface and endophytic and tightly bound cells associated with the adaxial leaf surface of basil increased initially and then decreased by day 14. On spinach, the concentration of epiphytic and tightly bound/endophytic cells of *V. cholerae* significantly decreased over time from the second time point onwards on the adaxial sides of the leaves; however, no epiphytic *V. cholerae* were recovered from the abaxial side of the leaves, whilst tightly bound and endophytic *V. cholerae* cells were only present at a low concentration at the first time point (Fig. 4 B and D).

**Fig. 5:**
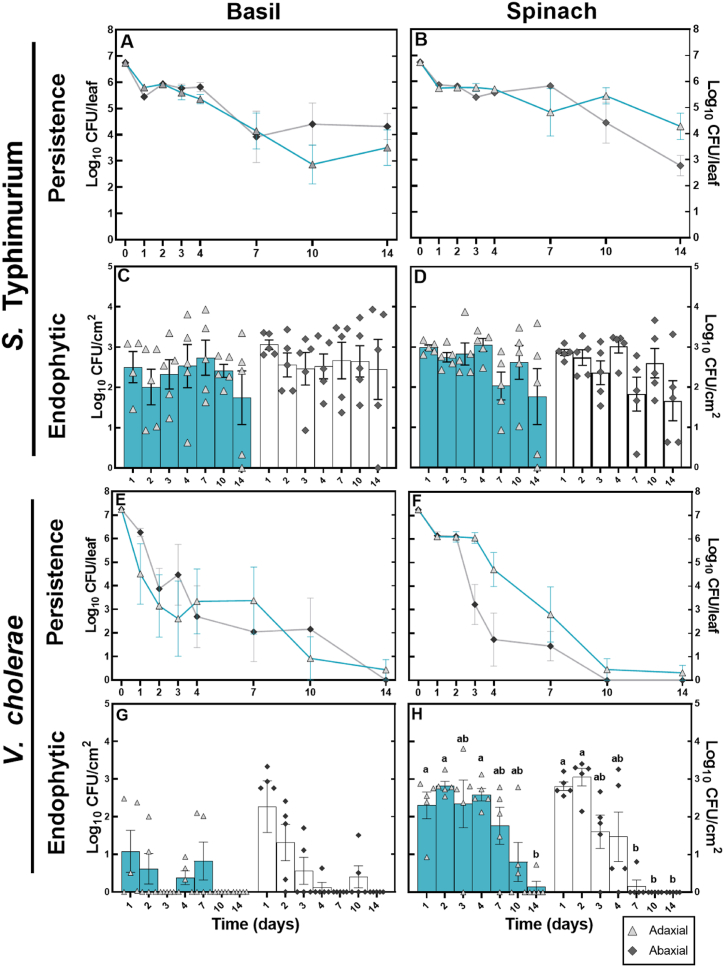
Concentration of *S*. Typhimurium and *V. cholerae* cells persisting on basil and spinach leaf surfaces, or tightly bound and endophytic, from the directly pipetted ‘culture-only’ controls. The x-axis represents the time since the plastic or hay fragments were placed on the plant leaf surface. All data is log transformed and represents the mean of five replicates ±SEM. Different letters above the bars indicate significant differences (P < 0.05). All concentrations of tightly bound or endophytic cells are reported as CFU/cm^2^, calculated from a 2.8 cm^2^ standardised disk cut from each leaf.

*S. typhimurium* directly inoculated onto basil and spinach leaves persisted for the 14-day period of the study (Fig. 5A and B), whilst the concentration of *V. cholerae* on basil leaves had nearly reached zero by day 14, and by day 10 on spinach leaves (Fig. 5E and F). Tightly bound and endophytic cells of *S.* Typhimurium were recovered on both the adaxial and abaxial leaf surfaces of basil and spinach at all timepoints (Fig. 5C and D), with no significant differences between the two plant species. On spinach, the concentration of tightly bound and endophytic cells of *V. cholerae* decreased significantly over time (P < 0.05) on both sides of the leaf (Fig. 5H). Tightly bound and endophytic cells of *V. cholerae* on basil were recoverable for up to 7 days on the adaxial surface and 10 days on the abaxial surface (Fig. 5G).

## 4 Discussion

### 4.1 Persistence of *S.* Typhimurium and *V. cholerae* in the plastisphere on plant surfaces

This study has demonstrated that the clinically and environmentally relevant pathogens, *S.* Typhimurium and *V. cholerae*, can persist in the plastisphere when adhered to the surface of leaves of two ready-to-eat crops. Importantly, both pathogens can dissociate from the plastisphere within 24 h of placement and become associated with the leaf surface, or even become tightly bound or internalised within the leaves. This highlights a clear food safety risk for those handling and consuming salad crops and herbs that are typically consumed raw, particularly in areas where agricultural plastic pollution is high.

Human enteric pathogens can use a pre-adaptive strategy, termed cross protection, to enhance their long-term survival in the environment, e.g., *Salmonella* spp. can adapt to a mild stress, which can enhance survival in harsher conditions [34,35]. In the plastisphere, enteric pathogens could be exposed to stressors such as UV light and desiccation (albeit at a lower level due to the protection afforded by the biofilm), and such adaptations may influence *S*. Typhimurium survival following dissociation from the plastisphere, before transfer into soils or onto the surface of crops (Woodford et al., 2024, unpublished). Despite the inherent differences between fragments of plastic mulch and hay, e.g., in terms of surface area, moisture content and availability of nutrients, *S*. Typhimurium was able to persist in the biofilm of both materials at relatively similar concentrations. However, initial increases in concentrations of *S*. Typhimurium on some of the fragments of hay (likely due to the provision of nutrients) indicate a transient benefit from being bound to an organic material. In contrast to organic materials, the durable nature of plastic polymers means that this material can persist in agricultural systems for much longer, with the potential for colonisation and re-colonisation by human pathogens [31].

The concentration of *Vibrio cholerae* recovered from the plastisphere rapidly declined within the first 3 days, but following enrichment cells were recovered at all the remaining timepoints up to day 14, suggesting that this pathogen had entered a viable but non-culturable (VBNC) state in the plastisphere [36]. Loss of culturability of pathogens such as *V. cholerae* can, in some cases, be attributed to the integration of host cells into a quorum-regulated biofilm [37]. Ready to eat crops contaminated with microplastics colonised by *V. cholerae* will likely be washed after being harvested, with the potential for any VBNC *V. cholerae* cells to become resuscitated in the water [30]. In this scenario, *V. cholerae* could enter a highly planktonic form in the water being used to wash crops; as *V. cholerae* often enters a dormant state in water but with periodic resuscitation [38], there is the potential for it to contaminate any plant leaves or other microplastics that it comes into contact with during this process. Unlike in the plastisphere, *V. cholerae* did not appear to enter the VBNC state when associated with the hay biofilm, and even increased in concentration during the first 72 h. As with *S*. Typhimurium, this is likely due to the pathogen utilising labile nutrients available on the surface of the hay [39].

### 4.2 Dissociation from the plastisphere and binding to the leaves of spinach and basil crops

Both *S.* Typhimurium and *V. cholerae* were able to readily dissociate from the plastisphere within 24 h of placement on leaf surfaces, with *S*. Typhimurium still detectable on the leaf after 14 days. This rapid dissociation means that contaminated plastics do not need to be in contact with plant surfaces for long to transmit pathogens onto their surfaces, and that their removal by washing or mechanical action when crops are harvested and prepared would not necessarily be effective at preventing pathogen incorporation into the phyllosphere, particular as in some cases washing of salad leaves like spinach does not significantly reduce the concentration of bacteria [40]. It is likely that the fragments of plastic and hay on the leaf surfaces provided a degree of protection from desiccation and UV by trapping moisture and blocking direct light exposure. Black specks were observed on some of the leaves in this study around the positions of the plastic or hay, and it has previously been reported that *S. enterica* on tomato plant leaves is associated with higher incidences of speck lesions on tomato plants [41].

The survival of *E. coli* and *Salmonella* spp. on ready-to-eat leaves is influenced by surface roughness, stomatal density, and the wax content of the leaves, although this varies depending on plant genotype [[42], [43], [44]]. It is likely that *S.* Typhimurium forms aggregated biofilms on the surface of both basil and spinach, given that a large proportion of the epiphytic bacterial population on leaves are located in large aggregates, typically in the veins and grooves of the leaf surface [45]. Epiphytic phyllosphere bacteria use cross-talk via quorum-sensing signals to communicate, including bacterial production of Acyl-homoserine lactones (AHLs) which can upregulate survival determinant factors, which may benefit human enteric pathogens incorporated within these populations [39]. Water soaking on the leaf surface (e.g., through the action of bacterial plant pathogens) can also promote survival of *S. enterica* [46], and the moisture contained within the Petri dishes housing the excised leaves in this study, and any moisture trapped between the microplastic fragments and the leaf surface, may have contributed to the enhanced survival of *S.* Typhimurium on the excised leaves.

Unlike *S.* Typhimurium, the survival of epiphytic *V. cholerae* following transfer from the plastisphere was dependent on crop type, with colonies detected at every time point on the adaxial and abaxial leaf surfaces of basil, but not on the abaxial surface of spinach leaves. *V. cholerae* can readily form biofilms in both fresh- and salt-water, which are considered to be the environmental reservoir for *V. cholerae* between epidemics due to the long-term viability in such biofilms [36,38]. *V. cholerae* is also thought to use quorum sensing as an adaptive process in different environments, whereby an accumulation of bacterial cells results in the expression of autoinducers which alter bacterial gene expression in an adaptive manner [47]. Directly pipetting *V. cholerae* suspension onto the leaf surface led to a rapid decrease in concentration of cells over a 14-day period indicating that *V. cholerae* did not readily form a biofilm on the plant leaf surface (and/or rapidly entered a VBNC state). However, these culture-only samples were added to the leaf surface at relatively high concentrations (∼10^7^ CFU/leaf) and due to transcriptional repression of genes which encode synthesis machinery, *V. cholerae* biofilm formation decreases at high cell density [47], offering a possible explanation for the rapid decrease observed here. Other factors, including a lack of availability of nutrients and the presence of other bacteria to aid biofilm formation may have also been factors in the rapid decrease in ‘culture only’ concentrations in this study.

### 4.3 Transfer from the plastisphere to the phyllosphere – implications for food safety and human health

In this study, both epiphytic and loosely bound, and tightly bound and endophytic cells of *S.* Typhimurium were detected on leaves of basil and spinach with concentrations remaining similar throughout the study. This indicates an initial process of transfer during the first 24 h of contaminated material coming into contact with the leaf, followed by persistence of the pathogens associating with the leaf. In basil, the stomata close and reduce in size under water deficit conditions, particularly on the adaxial leaf surface [48] and the adaxial leaf surface can also provide a harsher environment for pathogen survival, with UV exposure and desiccation likely to be stress factors [16]. As such, pathogens tend to colonise around veins on the leaf and move towards stomata, for increased protection and for subsequent internalisation [11,41,49]. In amphistomatic plants, stomata openings between the adaxial and abaxial surface of leaves, can be differentially induced by environmental stimuli; however, despite all these differences between adaxial and abaxial surfaces there was no significant difference in the concentration of *S*. Typhimurium recovered from either leaf surface for basil in this study. Microplastics can be difficult to remove from leaf surfaces [50], which could facilitate the potential for transfer from the plastisphere to the leaf surface. Pathogen internalisation via stomata poses an additional health risk as removal of plastic contaminants and epiphytic bacteria by extensive washing in clean water would not be sufficient to remove *S.* Typhimurium from basil or spinach before consumption [18].

No *V. cholerae* was detected on the abaxial side of spinach leaves following the adherence of hay fragments; however, *V. cholerae* cells were detected internally in the ‘culture-only’ control samples. It is possible that other bacteria in the hay biofilm community actively inhibited *V. cholerae* uptake on the spinach abaxial leaf surface or triggered stomatal closure [51]. Although some bacteria can produce toxins that impair stomatal function [52], *Salmonella* spp. antigens are not well recognised by the stoma-based plant immune response, allowing *Salmonella* cells to more readily enter the leaf [53]. Unlike *S.* Typhimurium, *V. cholerae* persistence significantly decreased over time, suggesting that it lacked the sufficient nutrients to survive, or was outcompeted in the phyllosphere, or was not able to overcome some aspect of innate plant immunity.

Internalisation of both *S.* Typhimurium and *V. cholerae* can also be facilitated by damage or degradation to the leaves. After leaves have been excised, they begin to senesce with a greater potential for colonisation by saprophytes; natural senescence together with saprophytic activity will increase the availability of nutrients for pathogens [11]. This may offer an enhanced mode of uptake into leaves for *S.* Typhimurium or *V. cholerae*, however in this study the concentrations of internalised pathogens did not change significantly over time, indicating that plant lesions were not a significant factor in pathogen internalisation. As plastics are able to persist for long periods of time in the environment, and strongly bind to plant surfaces [50], they could be increasing the risk of transfer of pathogens from the plastisphere. If for example, crops contaminated with microplastic fragments are damaged during harvesting processes, pathogens could dissociate from the plastisphere and enter crops via lesions within 24 h of harvesting.

### 4.4 Conclusion

The ability of *S.* Typhimurium and *V. cholerae* to survive in the plastisphere whilst adhered to plant leaves, and then subsequently dissociate from the plastisphere onto ready-to-eat crops has significant implications for food safety and public health. The rapid dissociation of human pathogens from the plastisphere, within 24 h of being placed on the plant leaf surface, provides a risk for handling and consumption of ready-to-eat crops. As plastics are lightweight and durable and readily used in agricultural processes, there is a risk of those pathogens associated with the plastisphere coming into contact with humans and livestock via contaminated edible plants. This could present a major health risk, as simple washing and good hygienic practice will not necessarily remove these pathogens before consumption.

## Data availability

Data will be made available by request.

## CRediT authorship contribution statement

**Luke Woodford:** Writing – review & editing, Writing – original draft, Formal analysis, Data curation, Conceptualization. **Rosie Fellows:** Writing – review & editing, Data curation. **Hannah L. White:** Writing – review & editing, Data curation. **Michael J. Ormsby:** Writing – review & editing, Data curation. **Richard S. Quilliam:** Writing – review & editing, Funding acquisition, Conceptualization.

## Declaration of competing interest

The authors declare that they have no known competing financial interests or personal relationships that could have appeared to influence the work reported in this paper.
