# Supplementary material for: Salmonella Typhimurium and Vibrio cholerae can be transferred from plastic mulch to basil and spinach salad leaves
Source: Heliyon. 2024 May 18;10(10):e31343. doi: 10.1016/j.heliyon.2024.e31343 (PMC11137414; doi:10.1016/j.heliyon.2024.e31343)
Supplement: Multimedia component 1 [file mmc1.docx]

Supplementary Files for *Salmonella* Typhimurium and *Vibrio cholerae* can be transferred from plastic mulch to basil and spinach salad leaves.

**
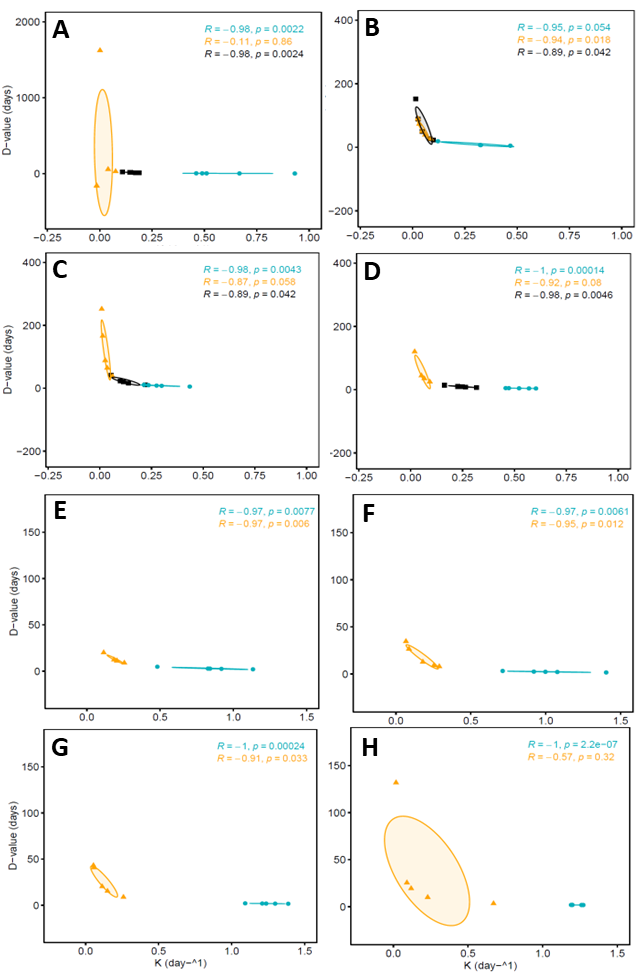
**

**Fig S1.** Correlation analysis of linear decline rates of *S*. Typhimurium (A-D) and *V. cholerae*

(E-H) survival on plastic, hay, and in culture on basil adaxial surface (A & E), basil abaxial surface (B & F), spinach adaxial surface (C & G) and spinach abaxial surface (D & G). Plastic samples are shown as black squares, hay are orange triangles, and culture-only controls are turquoise circles. The K-value and D-value from each linear decline analysis were plotted against each other for each plant type and leaf surface using a Pearson correlation coefficient (with the R score indicating the positive or negative correlation, P-value the significance of the correlation and the ellipse indicating the 95% confidence interval). Plot A is on a different scale from plots B-D due to one outlier in the hay data.
